# Evaluation of the Anti-apoptotic and Anti-cytotoxic Effect of Epicatechin Gallate and Edaravone on SH-SY5Y Neuroblastoma Cells

**DOI:** 10.32598/bcn.9.10.1159.2

**Published:** 2019-11-01

**Authors:** Mohammad Shokrzadeh, Hashem Javanmard, Golpar Golmohammad Zadeh, Hossein Asgarian Emran, Mona Modanlou, Saeed Yaghubi-Beklar, Ramin Ataee

**Affiliations:** 1. Pharmaceutical Sciences Research Center, Mazandaran University of Medical Sciences, Sari, Iran.; 2. Thalassemia Research Center, Mazandaran University of Medical Sciences, Sari, Iran.; 3. Immunology Research Center, Mazandaran University of Medical Sciences, Sari, Iran.

**Keywords:** Epicatechin, Edaravone, Apoptosis, SH-SY5Y, 6-OHDA, Neurodegenerative disorder, Parkinson disease, Neuroprotection, MTT

## Abstract

**Introduction:**

Parkinson disease (PD) is the second most common neurodegenerative disease affecting older individuals with signs of motor disability and cognitive impairment. Epicatechin (EC) and edaravone have neuroprotective effects most probably due to their antioxidant activity; however, a limited number of studies have considered their role in PD. This research aimed at investigating the neuroprotective effect of EC and edaravone in a neurotoxin-induced model of PD.

**Methods:**

An in vitro model of PD was made by subjecting SH-SY5Y neuroblastoma cells to neurotoxin: 6-hydroxydopamine (6-OHDA) 100 μM/well. The cytoprotective effect of EC and edaravone in five concentrations on cell viability was tested using the MTT (3-(4,5-Dimethylthiazol-2-yl)-2,5-Diphenyltetrazolium Bromide) assay. The apoptotic assay was done by annexin V and propidium iodide method using flow cytometry.

**Results:**

According to the MTT assay analysis, EC and edaravone had protective effects against 6-OH DA-induced cytotoxicity in SH-SY5Y neuroblastoma cells that were much more significant for edaravone and also a relative synergistic effect between EC and edaravone was observed. The apoptotic analysis showed that edaravone alone could decrease early and late apoptosis, whereas EC diminished early apoptosis, but enhanced late apoptosis and necrosis. Besides, co-treatment of edaravone and EC had a synergistic effect on decreasing apoptosis and increasing cell viability.

**Conclusion:**

The protective effect of edaravone on apoptosis and cytotoxicity was demonstrated clearly and EC had a synergistic effect with edaravone.

## Highlights

- Epicatechin (EC) and edaravone have neuroprotective effects most probably due to their antioxidant activity;
- According to this research, MTT assay analysis shown, EC and edaravone had protective effects against 6-OH DA induced cytotoxicity in SH-SY5Y neuroblastoma,
- Protective effect was much more significant for edaravone and also a relative synergistic effect between EC and edaravone was observed.
- The apoptotic analysis showed that edaravone alone could decrease early and late apoptosis, whereas EC diminished early apoptosis, but enhanced late apoptosis and necrosis
- EC had a synergistic effect with Edaravone on decreasing apoptosis and increasing cell viability.

## Plain Language Summary

Parkinson Disease (PD) is the second most common neurodegenerative disease affecting older individuals with signs of motor disability and cognitive impairment. Epicatechin (EC) and edaravone have neuroprotective effects most probably due to their antioxidant activity; however, a limited number of studies have considered their role in PD. This research aimed at investigating the neuroprotective effect of EC andedaravone in a neurotoxin-induced model of PD.Our study on cell proliferation and apoptosis in a cell model of parkinson diseases shown that both EC and Edaravone have cytoprotective effect and antiapoptotic effect which this effect for Edaravon was more potent and there was synregism effect between Edaravone and epichatechine

## 1. Introduction

Parkinson Disease (PD) is an age-dependent cumulative neurodegenerative illness that is estimated to affect almost 9 million over 50 years old people by 2030 (Dorsey et al., 2007). Pathologically, PD occurs due to the loss of dopaminergic neurons in the substantia nigra, which in turn induces dopamine depletion in the striatum (Dauer & Przedborski, 2003). An abnormal accumulation of alpha-synuclein named Lewy bodies is also diagnosed in surviving neurons (Przedborski, 2005). Dopamine loss finally can lead to disturbing more motor functions, resulting in clinical signs in patients, such as tremor, rigidity, and slow responsiveness (Snyder & Adler 2007). Several causes, such as neuroinflammation, mitochondrial dysfunction, failure of the ubiquitin-proteasome system, and proteinopathy, have been proposed to describe the neurodegeneration events in PD (Schulz, 2007), among which oxidative stress-related apoptosis has been involved in the pathogenesis of neurodegenerative diseases. Oxidative stress is produced by the accumulation of excessive partially reduced Reactive Oxygen Species (ROS) within the cell, which attack electron-rich biological molecules, such as DNA, proteins, and lipids affecting cellular functions (Wang, Xie, Wang, & Bi, 2014). ROS is produced as a part of normal cellular oxidative materials that damages neuronal biomolecules (Gandhi & Abramov, 2012) and increases the mass of iron in specific areas of the brain and is considered as the major pathological aspects of PD and Alzheimer Disease (AD) (Riederer, et al., 1989).

Generally, the disturbance of the equilibrium between pro-oxidant and antioxidant homeostasis leads to oxidative stress that can further produce ROS in neuronal cells (Lepoivre, Flaman, Bobé, Lemaire, & Henry, 1994). Post-mortem studies have shown that in PD, mitochondrial function impairment and ROS accumulation are two events associated with apoptotic pathways in dopaminergic neurons (Yew, Koh, Chye, Othman, & Ng, 2014). Also, post-mortem examinations have revealed that nigral cell death in PD is related to an increase in lipid peroxidation (Dexter et al., 1989), decreased reduced glutathione (GSH) levels (Sofic, Lange, Jellinger, & Riederer, 1992), enhanced superoxide activity (Saggu et al., 1989) and increased levels of iron in substantia nigra (Dexter et al., 1987). It has been reported that mitochondrial injury may have a key role in the pathogenesis of AD and PD (Du et al., 2010) and several studies have shown that various xenobiotics can increase the risk of PD and AD via mitochondrial dysfunction or oxidative damage (Agnati et al., 2005; Cuevas et al., 2009).

Therefore, mitochondrial protection and subsequent reduction of oxidative damage can be considered as a therapeutic strategy to cure these diseases (Ataie, Sabetkasaei, Haghparast, Moghaddam, & Kazeminejad, 2010; (Du et al., 2010). Although the exact mechanism of nigral cell death in PD is still unknown, oxidative stress is strongly considered as an essential factor. Dopamine replacement with levodopa or dopamine agonists, like madopar is currently the main drug for the treatment of PD (Jenner & Olanow 1996). Although these drugs are effective in the early stages of PD, they may be associated with serious adverse effects in the long-term. Therefore, a therapeutic strategy for PD treatment may act via modulation of oxidative stress (Birkmayer et al., 1985), which can result in a better prognosis and treatment.

Phenolic compounds with antioxidant properties, such as flavonoids, are a large group of natural products that are widely distributed in plants and are the main constituents of many fruits, nuts, leaves, etc. (Kühnau, 1976). Green tea has protective effects against neurological diseases, such as PD, AD, and ischemic damages. It also has an anti-diabetic effect in insulin-resistant animal models. Besides, other properties of green tea, such as antibacterial, anti-HIV, and anti-aging effects, have been reported (Sutherland, Rahman, & Appleton, 2006). The antioxidant effect of green tea is due to its polyphenols, like catechin and its ability to scavenge ROS, such as hydroxy-phenol groups on the B-ring of non-galolite of Epicatechin (EC) and Epigallocatechin (EGC), B-ring and D-ring of galolite of epicatechin-3-gallate and Epi-Gallocatechin Gallate (EGCG).

The presence of 3, 4, 5-trihydroxy B-ring is important for antioxidant and radical scavenging properties of catechins. The antioxidant effects of catechins in green tea is more than vitamin C and E (Sutherland et al., 2006). Flavonols, such as (−)-EC, represent a major class of flavonoids commonly present in some plants, such as Camellia sinensis (green tea) (Cuevas et al., 2009). Moreover, studies on rats using EC extracts have demonstrated some effectiveness regarding oxidative stress, cognitive function, and memory performance (Shah et al., 2010). Green tea polyphenols have also been confirmed to hinder the apoptotic pathway of mitochondria (increasing Bcl2 and decreasing caspase-3 activity), protect mitochondrial membrane potential, inhibit ROS production, and regulate calcium concentration levels (Guo, Bezard, & Zhao, 2005).

Amyloid-beta neurotoxicity has been associated with producing free radicals, which can be prevented by effective radical scavengers (Christen, 2000). More effective radical scavengers have been shown as good amyloid beta inhibitors (Ghosh, Pandey, & Dasgupta, 2013). The radical scavenging specifications of GTPs are as order as ECG>EGCG>EGC (Kondo, Kurihara, Miyata, Suzuki, & Toyoda, 1999; Zaveri, 2006) that indicates a better inhibitory activity of ECG than the two others. According to this evidence, specific attention has been focused on studying the antioxidants’ neuroprotective effect, iron chelating and anti-inflammatory qualities of tea flavonoids, especially (−)-epicatechin-3-gallate (ECG) (Slikkker et al., 1999).

The novel free radical scavengers are considered based on their neuroprotective activity, including calcium homeostasis (Ishige, Schubert & Sagara, 2001), the extra-cellular Mitogen-Activated Protein Kinases (MAPK) (Schroeter et al., 2002), protein kinase C (PKC) (Levites, Amit, Mandel & Youdim, 2003), antioxidant enzymes (Levites, Weinreb, Maor, Youdim, & Mandel, 2001), Antioxidant Regulatory Element (ARE) (Chen, Yu, Owuor, & Kong, 2000), survival genes (Levites, Amit, Youdim, & Mandel, 2002), and the Amyloid Precursor Protein (APP) pathway (Levites et al., 2003). Edaravone, as a novel free radical scavenger, has been used recently for inhibiting oxidative stress and inducing apoptosis in some diseases. It is the first clinical drug for neuroprotection, used for ischemic stroke since June 2001 and has shown effectiveness on cerebral injury in ischemic situations (Kikuchi et al., 2009). Edaravone blocks neuronal damage by reducing microglia-derived free radicals and nitric oxide (Kaur & Ling, 2008; Yan et al., 2012). Edaravone is a drug with antioxidant effect that decreases hydroxyl radicals and superoxide radical production (Ito et al., 2008). In addition, it has recently showed neuroprotective properties in neurodegenerative diseases, such as PD, AD, and amyotrophic lateral sclerosis (Xiong et al., 2011).

Edaravone has antioxidant and anti-apoptotic properties that can also block lipid peroxidation, maintains mitochondrial function, and provides energy (Zhang et al., 2013). It decreases ROS production and induces MAPK signaling pathway activation which protect HT22 cells against H2O2 toxicity (Zhao et al., 2013). It can protect dopaminergic neurons against neurotoxicity with rotenone and 6-hydroxydopamine (6-OHDA) and blocks apoptosis in the dopaminergic neurons, alleviates ROS production, down-regulates Bax expression and up-regulates VMAT2 expression (Yuan et al., 2008; Xiong et al., 2011). In 6-OHDA-induced PD models in vitro and in vivo, edaravone is neuroprotective for dopaminergic neurons (Yuan et al., 2008). Therefore, edaravone is effective for the treatment of PD. However, further investigations are needed to find the mechanism underlying its neuroprotection properties. The present study aimed at evaluating the synergistic effect of epicatechin and edaravone in combination with levodopa as anti-Parkinson drug (L-Dopa;) on the proliferation and apoptosis in SH-SY5Y neuroblastoma cell line affected by 6-OH Dopamine as an in vitro PD model using MTT assay and flow cytometry method.

## 2. Methods

### 2.1. Drugs and reagents

Edaravone, 6-OHDA, ECG, Dulbecco’s modified Eagle’s medium (DMEM), and fetal bovine serum (FBS) were purchased from the Sigma Chemical Co. (Sigma Aldrich, Germany). Levodopa was prepared from the Roach and Sigma companies (Switzerland). Penicillin and streptomycin were purchased from the Roach, and also SHSY5Y neuroblastoma cell line was provided from the National Cell Bank of Iran (Pasteur Institute of Iran).

### 2.2. Cell culture

SH-SY5Y cells were cultured in DMEM and supplemented with 10% FBS, penicillin (100 IU/mL) and streptomycin (100 μg/mL). The medium was refreshed every 2 days. Cell cultures were kept at 37° C in a humidified atmosphere containing 95% air and 5% CO (Yew et al., 2014).

### 2.3. Cell viability assay for SH-SY5Y cells

Cell viability was assessed by MTT assay, which measures the activity of mitochondria, based on the reduction of yellow tetrazolium salt to purple formazan by the dehydrogenase activity of mitochondria. First, the cells were washed once with PBS before adding 0.1 mL serum-free medium containing MTT (1 mg/mL) to each well. Then they were incubated for 3 h, the supernatant was removed, and the obtained formazan product was dissolved in 1 mL of dimethyl sulfoxide (DMSO) with stirring for 15 min on a microtiter plate shaker, and the absorbance was detected at 550 nm. The percentage of viable cells in each treatment group was determined by comparing their respective absorbance with the control group (Yew et al., 2014).

### 2.4. Drug treatments

Different concentrations of ECG, edaravone, and levodopa (30, 60, 125, 250, and 500 μg/mL) were incubated for 1 h into SH-SY5Y cells, which were split in the 96-well plates at a density of 1.0×104 /well (IC50 concentration was calculated for each of these materials) (Yew et al., 2014). They were then exposed to 100 μM of 6-OHDA for 24 h in 96-well plates. Besides, 6-OHDA was considered as a positive control, whereas the DMEM was a negative control. After a 48-h incubation, the MTT solution was added into the culture to a final concentration of 0.5 mg/mL. Four hours later, the medium, which was kept at 37°C, was replaced with an equal volume of DMSO and dissolved in the purple formazan crystal. Then, the absorbance was measured spectrophotometrically with a microplate reader (Dynex Opsys MR 24100) at 570 nm and compared with the control (Yew et al., 2014).

### 2.5. Apoptosis analysis (flow cytometry)

Apoptosis procedure was assessed by the Annexin VFITC Apoptosis Detection Kit (BD Pharmingen, USA). Briefly, after the treatments (incubating the cells with different drugs for 24 h in a CO _2_ incubator at 37° C), the cells were harvested and washed with binding buffer. Then, the cells were counted and a final concentration of 1×106 cells/mL was obtained. Annexin V and propidium iodide (PI) were then added and the cells incubated in the dark for 15 min. After washing, the cells’ suspension was fixed with 1% formaldehyde for 10 min on ice. After washing cells twice with binding buffer and adding RNAase enzyme (EMD Biosciences, USA), they were incubated for 15 min at 37° C. At last, the cells were washed and analyzed with FACSCalibur Flow Cytometer (BD Biosciences, USA) and the ProQuest software (Yew et al., 2014).

### 2.6. Statistical analysis

Data were analyzed in triplicate with at least 3 independent experiments. Values are expressed as Mean±SD. Statistical significance was examined by the Analysis of Variance (ANOVA) and dunnett t-test (P<0.05).

## 3. Results

### 3.1. In vitro assay

**Figure 1.**
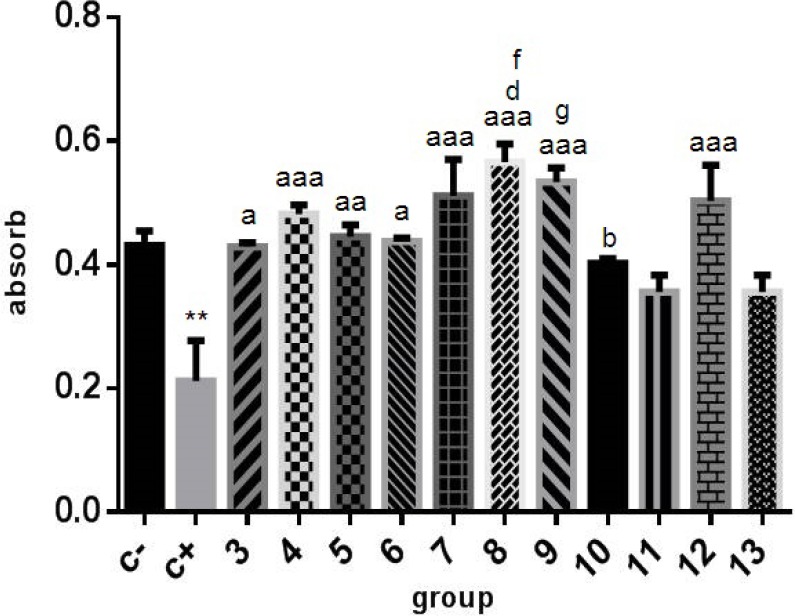
MTT assay The results of cell viability following different treatments on SH-SY5Y neuroblastoma cells after 24 h incubation. All cells were treated both with the drugs in their EC50 concentration and 100 μM of 6-OHDA; epicatechin (3) significantly increased the proliferation of neuroblastoma cells and this effect for edaravone (4) was more significant and also a synergistic effect between epicatechin, levodopa, and edaravone was observed (columns 7, 8, and 9). a: P<0.01 compared with the positive control (C+); aa: P<0.001 compared with the positive control (C+); aaa: P<0.0001 compared with the positive control (C+); b: P<0.05 compared with the positive control (C+); d: P<0.01 compared with column 11; f: P<0.01 compared with column 13; g: P<0.05 compared with column 11. 1: Negative control; 2: Positive control (6-OHD); 3: Epicatechin; 4: Edaravone; 5: Levodopa 1; 6: Levodopa 2; 7: Epi+levadopa1+edaravone; 8: Epi+levadopa2+edaravone; 9: Levadopa2+edaravone; 10: Epicatechin +levodopa 1; 11: Epicatechin+Levadopa 2; 12: Levadopa 1+edaravone; 13: Epicatechin without 6OHD

According to MTT assay, 6-OH DA as positive control significantly decreased cell viability compared with the negative control (Figure 1). EC at EC50 concentration had protective effect (column 3; P<0.01). The protective effect of edaravone at EC50 concentration was evenly much more significant (column 4; P<0.0001).

Levodopa (positive anti-Parkinson control), as expected, had a protective effect (columns 5 and 6; P<0.001). According to the graph, the protective effect of edaravone was even far higher than levodopa (column 4; P<0.0001). There was a partial synergistic effect between edaravone and ECG with levodopa (columns 7 and 8; P<0.0001), whereas the synergic effect between ECG and levodopa was not noticeable (columns 10 and 11; P<0.05). Also, ECG could increase the impact of edaravone (columns 7 and 8). Levodopa 1 was purchased from Roach Co; levodopa 2 was purchased from Sigma Co.

### 3.2. Apoptosis analysis

**Figure 2.**
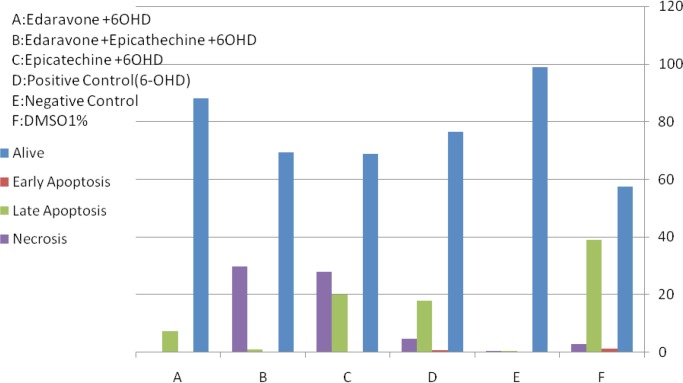
Apoptosis assay results with Annexin-V propidium iodide flow cytometry method of variant treatments on SH-SY5Y neuroblastoma cells following 24 h incubation

**Figure 3.**
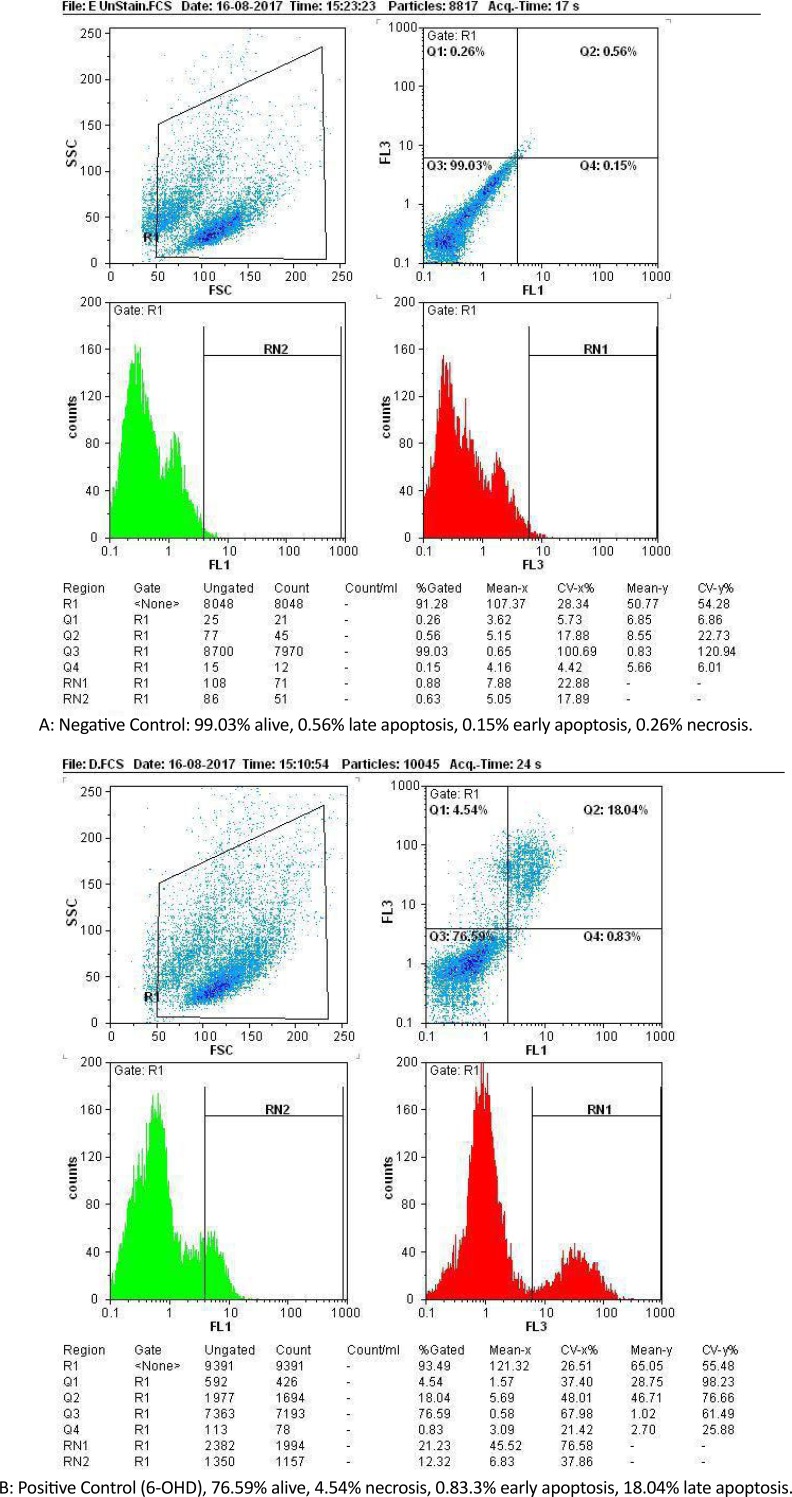

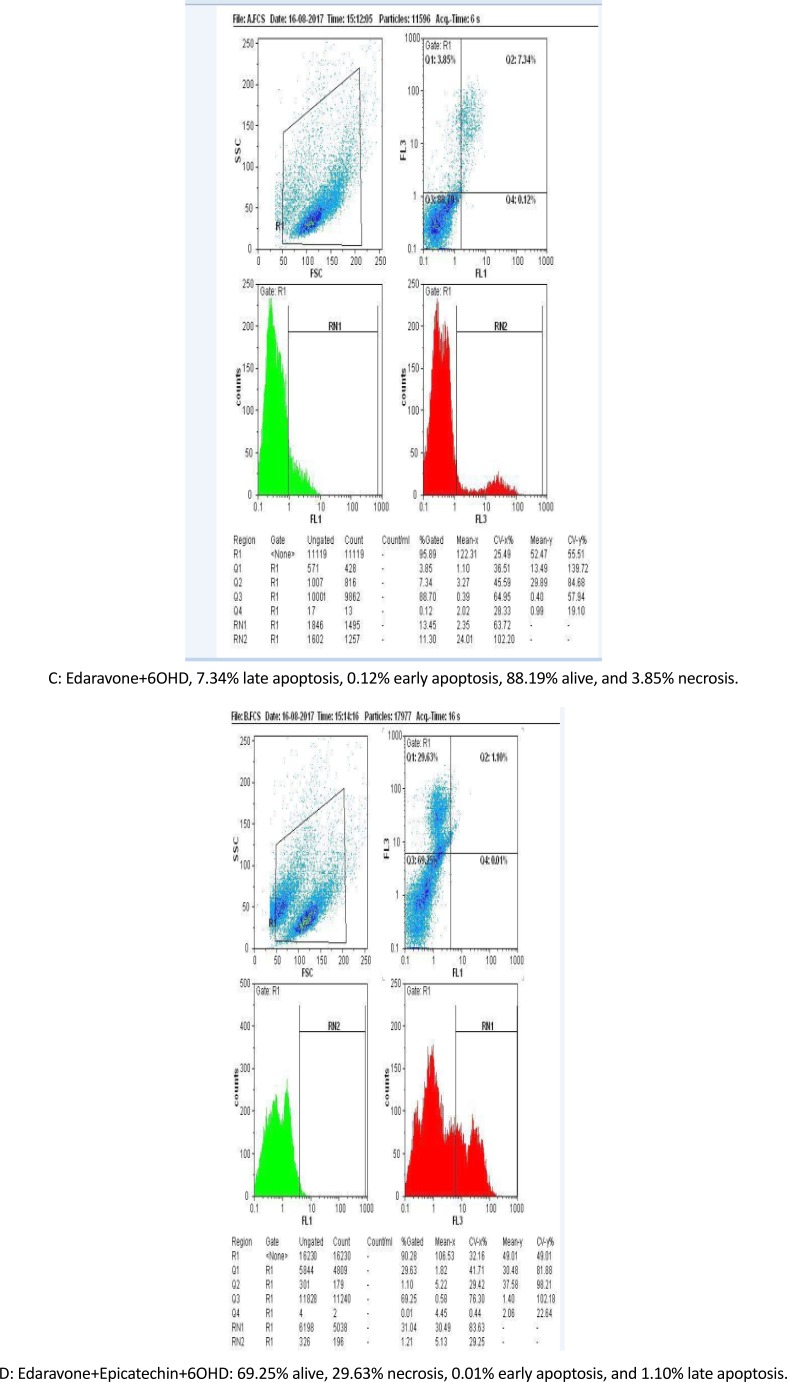

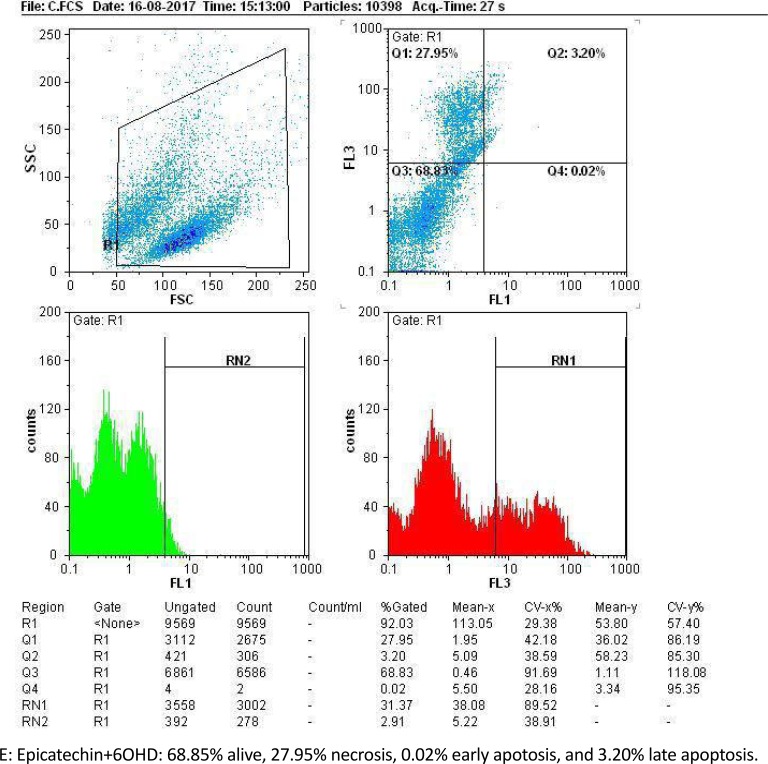
Fluorescence-Activated Cell Sorter (FACS) analysis of the cells following flow cytometry

Figures 2 and 3 are showing apoptosis assay results with Annexin-V propidium iodide flow cytometry method of variant treatments on SH-SY5Y neuroblastoma cells following 24 h incubation. According to the flow cytometry results, negative control samples had the highest levels of living cells (99%), and the rate of cell apoptosis and necrosis was very low (<0.5% Figure 3A). In positive control samples (6-OH DA), the rate of living cells drastically dropped (76%), whereas the rate of apoptosis (early and late) and necrosis significantly increased in comparison with the negative control (Figure 3 B). Using edaravone, early and late apoptosis decreased to 0.12% and 7.34%, respectively, which was comparable with the positive control (Figure 3 C). It also reduced evenly to 0.01% and 1.1% using edaravone and ECG synergistically (Figure 3 D) and 0.02%–3.2% for ECG alone (Figure 3 E). Therefore, both ECG and edaravone could prevent apoptosis, and a synergistic effect was observed between edaravone and ECG. It should be noticed that a decrease in apoptosis and necrosis is considered as the effectiveness of an anti-Parkinson drug.

## 4. Discussion

According to the obtained results, edaravone and ECG had anti-cytotoxic and anti-apoptotic effects in an in vitro model of PD in neuroblastoma cells, and this effect was more considerable for edaravone. In addition, a synergism effect was observed between edaravone and EC. Different neurotoxins, such as rotenone, 1-methyl-1,2,3,6-tetrahydropyridine (MPTP), 6-hydroxydopamine (6-OHDA), and paraquat have been used as experimental models of PD to imitate the neuropathology of this neurodegeneration both in vitro (i.e., human neuroblastoma SK-N-SH cells) and in vivo (animals models) (Hien, Gortnizka, & Kraemer, 2003).

6-OHDA as a catecholaminergic neurotoxin can cause an irreversible loss of nigrostriatal dopaminergic neurons by producing ROS and inhibiting complex I and complex IV of the electron transport chain through intra-cerebral infusion (Zuo & Motherwell, 2013). The selective disturbance of dopaminergic neurons in Substantia Nigra has shown to be the direct cause of neurodegeneration in PD (Olanow & Tatton, 1999). Also, it has been reported that 6-OHDA can cause dopaminergic cell death (Schober, 2004).

Green tea polyphenols can permeate into the brain (Suganuma et al., 1998; El Mohsen et al., 2002) and have a relatively potent metal-chelating efficacy (Guo, Zhao, Li, Shen, & Xin, 1996; Grinberg et al., 1997), which has been due to the gallate moiety present in the C-ring of both EGCG and ECG (Kumamoto, Sonda, Nagayama, & Tabata, 2001). Many studies on PD have revealed a moderate reduction in the risk of PD among tea users compared with non-drinkers (Weinreb, Mandel, Amit, & Youdim, 2004). Accumulation of iron at brain areas associated with neurodegeneration is lower in tea drinkers (Erikson, Shihabi, Aschner, & Aschner, 2002).

Green tea catechins decrease the incidence of cancer, arthritis, and UV damage in the skin (Pan, Jankovic & Le, 2003). It has been proved that EC and EGCG were more potent than catechin (Gómez-Guzmán et al., 2012). ECG has been reported to have the highest antioxidant activity compared with other tea polyphenols (Jin, Shen, & Zhao, 2001). Therefore, the efficacy of polyphenols to act as radical scavengers and chelators of transitional metals as iron and copper can be considered for the treatment of PD and AD (Weinreb,et al., 2004). Herbal medicines, such as ECG and chunghyuldan have currently been studied in neurodegenerative diseases, like AD and PD (Fernández-Moriano, González-Burgos, & Gómez-Serranillos, 2015). They have shown to inhibit apoptosis and ROS generation and maintain mitochondrial membrane potential (Kim et al., 2010). Among the isolated natural products, polyphenol has widely regarded. Resveratrol as another polyphenol compound, has also been shown with in vitro primary fibro-blasts cultures in PD patients who carry PARK2 mutations to regulate homeostasis of the mitochondrial energy due to an increase in complex I activity, citrate synthase activity, basal oxygen consumption, ATP production, and a decrease in lactate (Ferretta et al., 2014).

As a novel free radical scavenger, edaravone can inhibit oxidative stress and apoptosis (Watanabe, Tanaka, Watanabe, Takamatsu, & Tobe, 2004) with its antioxidant properties. Also, it can decrease the production of hydroxyl and superoxide radicals (Ito et al., 2008). Edaravone inhibits the production of Nitric Oxide (NO) and ROS through the activated microglia and protect meth-amphetamine-induced striatal dopaminergic neurotoxicity by peroxynitrite scavenging (Kawasaki et al., 2006). In addition, edaravone has been recently shown with neuroprotective efficacy in neurodegenerative diseases, such as PD, AD, and amyotrophic lateral sclerosis (Yan et al., 2012). It has in vitro and in vivo antioxidant and anti-apoptotic effects and also can inhibit lipid peroxidation, preserve mitochondrial function and energy supply, and reduce caspase activity (intrinsic and extrinsic pathways) (Zhao et al., 2013). Edaravone and EC can have a protective role on neurons (Kawasaki, Ishihara, Ago, Baba, & Matsuda, 2007; Mandel, Amit, Weinreb, Reznichenko, & Youdim,, 2008).

The present study confirmed the protective effect of edaravone and ECG against neurotoxicity in PD, which is consistent with the other studies indicating the protective role of these compounds in neurodegenerative diseases. ROS scavenging and antioxidant properties of these compounds can explain their protective effect in neurodegenerative diseases. Moreover, a synergic effect was found between EC and edaravone in reducing anti-cytotoxic and anti-apoptotic effects, whereas edaravone was more effective, and also EC when using alone showed a substantial impact. Also, we showed that levodopa co-treatment could increase its therapeutic effect in vitro synergistically.

## 5. Conclusion

ECG had protective effect in the apoptosis of nerve cells, and it increases the protective effect of edaravone when used as a co-treatment. Further investigations are necessary in animal models to identify and indicate the properties of these compounds precisely. In summary, the present study showed that EC and especially, edaravone could be considered as co-treatment in the therapeutic regime of PD.

## Ethical Considerations

### Compliance with ethical guidelines

All procedures were under ethical guideline of Deputy of research of Mazandaran university of medical Sciences with ethical Code of 1375.
